# Clozapine treatment and risk of COVID-19

**DOI:** 10.1192/bjo.2022.537

**Published:** 2022-07-11

**Authors:** Emanuele F. Osimo, Jonathan Lewis, Rudolf N. Cardinal, Golam M. Khandaker

**Affiliations:** Institute of Clinical Sciences, Faculty of Medicine, Imperial College Institute of Clinical Sciences and MRC London Institute of Medical Sciences, Hammersmith Hospital Campus, London, UK; Department of Psychiatry, University of Cambridge, UK; Adult Mental Health directorate, Cambridgeshire and Peterborough NHS Foundation Trust, Cambridge, UK; and South London and Maudsley NHS Foundation Trust, UK; Research and Development, Cambridgeshire and Peterborough NHS Foundation Trust, Cambridge, UK; Department of Psychiatry, University of Cambridge, UK; and Primary Care and Liaison Psychiatry, Cambridgeshire and Peterborough NHS Foundation Trust, Cambridge, UK; MRC Integrative Epidemiology Unit, Department of Population Health Sciences, Bristol Medical School, University of Bristol, UK; Centre for Academic Mental Health, Department of Population Health Sciences, Bristol Medical School, University of Bristol, UK; NIHR Bristol Biomedical Research Centre, UK; and Avon and Wiltshire Mental Health Partnership NHS Trust, UK

**Keywords:** Antipsychotics, psychotic disorders, schizophrenia, COVID-19, clozapine

## Abstract

The antipsychotic clozapine is known to have immune-modulating effects. Clozapine treatment has been reported to be associated with increased risk of COVID-19 infection. However, it remains unclear whether this is because of increased testing of this patient group, who are closely monitored. We linked anonymised health records from mental health services in Cambridgeshire (UK), for patients taking antipsychotic medication, with data from the local COVID-19 testing hub. Patients receiving clozapine were more likely to be tested for COVID-19, but not to test positive. Increased testing in patients receiving clozapine suggests prudent judgement by clinicians, considering the overall health vulnerabilities of this group.

Clozapine is one of the most effective antipsychotic medications, and the only medication licensed for treatment-resistant schizophrenia in the UK.^1^ In this population, clozapine can improve positive symptoms, hospital admissions and all-cause mortality.^2^ However, in a subgroup of patients clozapine treatment is associated with immune-mediated side-effects, notably neutropenia,^3^ requiring mandatory neutrophil count monitoring in most countries.^4^ Meta-analysis shows increased risk of COVID-19 mortality associated with psychotic disorder and exposure to antipsychotics.^5^ A previous study of National Health Service (NHS) electronic mental health records reported increased risk of COVID-19 infection in patients with schizophrenia who received clozapine.^6^ It has been speculated that the immune-modulating effects of clozapine might account for this association. However, it remains unclear whether such patients are more likely to be tested for COVID-19 in the first place, given the close clinical monitoring offered to this patient group. It also remains unclear whether patients with schizophrenia taking clozapine are more likely to test positive for COVID-19 after considering testing frequency.

## Method

We linked de-identified electronic health records of the secondary-care NHS mental health provider in Cambridgeshire, Cambridgeshire and Peterborough NHS Foundation Trust (CPFT), with one of the two local COVID-19 testing hubs (Cambridge University Hospitals NHS Foundation Trust; CUH). COVID-19 testing data included all molecular (polymerase chain reaction; PCR) tests performed at the CUH in the given time frame. We investigated whether, compared with patients treated with other antipsychotics, patients receiving clozapine were more likely to be tested for COVID-19; if tested, were more likely to test positive for COVID-19; and were more likely to receive a positive COVID-19 test result overall (irrespective of the rate of testing). We compared patients treated with clozapine with those treated with any other antipsychotic medication. Two patient groups were made: patients treated at any point over an approximately 7-year period (from 1 January 2013 to 30 April 2021) and patients treated ‘currently’ (from 1 May 2019 to 30 April 2021). Patients were selected through an electronic search of the CPFT Research Database.^7^ ‘Exposure to antipsychotics’ was operationalised as at least two mentions of the same British National Formulary class antipsychotic, at least 1 month apart. Patients receiving clozapine were identified by matching CPFT Research Database records with the CPFT clozapine database. Logistic regression was used to calculate odds ratios and 95% confidence intervals for COVID-19 outcomes, adjusting for age, gender and ethnicity.

The authors assert that all procedures contributing to this work comply with the ethical standards of the relevant national and institutional committees on human experimentation and with the Helsinki Declaration of 1975, as revised in 2008. All procedures involving human patients were approved by the UK NHS Research Ethics Service (references 12/EE/0407 and 17/EE/0442); linkage to COVID-19 test data was obtained, in de-identified fashion, via the EpiCov project (CUH) (CPFT project reference M00997). See Supplementary material available at https://doi.org/10.1192/bjo.2022.537 for further information on methods.

## Results

A total of 13 726 patients were prescribed an antipsychotic during the study period (January 2013 to April 2021) and were included in the study. Of these, 1943 (14%) had a COVID-19 test, 2123 (15.5%) had a coded diagnosis of schizophrenia and 778 (6%) were prescribed clozapine.

The odds ratio for patients receiving clozapine being tested for COVID-19 was 1.99 (95% CI 1.70–2.33, *P* < 2×10^–16^) versus patients on other antipsychotics, after adjusting for age, gender and ethnicity. After adding a coded diagnosis of schizophrenia as a predictor, the odds ratio reduced to 1.32 (95% CI 1.10–1.59, *P* = 0.002) (Supplementary Table 1).

Among patients tested for COVID-19, clozapine use was associated with an odds ratio of 1.22 (95% CI 0.68–2.07, *P* = 0.48) for a positive test result, after adjusting for age, gender and ethnicity. The odds ratio adjusted further for a coded diagnosis of schizophrenia was 1.06 (95% CI 0.56–1.91, *P* = 0.84) (Supplementary Table 2).

Finally, the odds ratio for patients receiving clozapine (versus others) having a positive test for COVID-19 irrespective of testing rates was 2.09 (95% CI 1.18–3.48, *P* = 0.007), after adjusting for age, gender and ethnicity. The odds ratio further adjusted for a coded diagnosis of schizophrenia was 1.25 (95% CI 0.67–2.26, *P* = 0.46) (Supplementary Table 3).

Sensitivity analyses of 13 268 patients ‘currently’ prescribed antipsychotic medications yielded similar findings. Patients receiving clozapine had higher odds of being tested for COVID-19, but not higher odds of testing positive if tested. They had higher odds of testing positive when testing frequency was not accounted for, although the 95% confidence interval for this estimate included the null hypothesis, possibly because of the lower sample size (Supplementary Tables 1–3).

## Discussion

Our findings suggest that, compared with secondary healthcare patients treated with other antipsychotics, patients receiving clozapine were more likely to be tested for COVID-19, but they were not more likely to test positive if tested. However, our results also show that patients receiving clozapine were more likely to test positive for COVID-19 irrespective of the difference in testing rates, if a schizophrenia diagnosis was not taken into account.

Our findings are compatible with previous work,^6^ and extend it in various ways. We show that the potential association between clozapine treatment and an increased risk of testing positive for COVID-19 could, at least in part, be attributed to increased rates of testing in this patient group, who are under greater surveillance, and appropriately so. Furthermore, we use two sets of complementary analyses based on either patients treated with antipsychotics over a longer 7-year period or current treatment limited to the latest 24 months, yielding compatible findings. Limitations of the work include reliance on electronic health record data, which depends upon clinicians recording information and thus is prone to missing information, a common problem for electronic health record-based studies. For example, schizophrenia was likely significantly undercoded in this data-set, rendering tentative conclusions about diagnosis. Our study period captures the first 12 months of the COVID-19 pandemic in the UK, and so may not be generalisable to other countries or time frames. Further, COVID-19 testing data did not include community testing results, or tests carried out at the other Cambridgeshire hospital (Peterborough City Hospital); therefore, the EpiCov data-set included all emergency department and in-patient tests performed at the CUH, covering approximately half of CPFT's catchment area by population. Finally, PCR test availability for SARS-CoV-2 varied considerably over time: initially only available for in-hospital testing of high-risk cases, it became gradually available for home self-testing of any symptomatic member of the public over the course of the pandemic. However, we do not believe that availability would have differentially affected secondary healthcare patients treated with other antipsychotics versus patients receiving clozapine.

We conclude that increased testing for COVID-19 in patients receiving clozapine, as observed in our results, shows prudent clinical judgement by clinicians and should continue, considering the notable health vulnerabilities of this patient group, which include multimorbidity and the immune-mediated effects of clozapine.

## Data Availability

Conditions of the ethics application of both the EpiCov database and CPFT Research Database mean that the anonymised data-sets are only available to local employees following an ethics application. Code is available on request to the corresponding author, E.F.O.

## Supplementary material

For supplementary material accompanying this paper visit https://doi.org/10.1192/bjo.2022.537. click here to view supplementary material
