# Supplementary material for: Clozapine treatment and risk of COVID-19
Source: BJPsych Open. 2022 Jul 11;8(4):e131. doi: 10.1192/bjo.2022.537 (PMC9273724; doi:10.1192/bjo.2022.537)
Supplement: Supplementary file 1 [file S2056472422005373sup001.docx]

Clozapine treatment and risk of COVID-19

Osimo et al, 2022

Supplementary Materials

Table of contents:

[Supplementary Methods 2](#_Toc101967778)

[Supplementary Table 1: Were clozapine-treated patients more likely to be tested for COVID-19, as compared with other antipsychotic-treated patients? 4](#_Toc101967779)

[Supplementary Table 2: If tested for COVID-19, were clozapine-treated patients more likely to test positive compared with other antipsychotic-treated patients? 5](#_Toc101967780)

[Supplementary Table 3: Were clozapine-treated patients more likely to test positive compared with other antipsychotic-treated patients, irrespective of testing rates? 6](#_Toc101967781)

## Supplementary Methods

An electronic search of the CPFTRD (UK National Health Service [NHS] Research Ethics Service references 12/EE/0407, 17/EE/0442), which contains de-identified electronic health records (EHRs), was used to select all patients who were prescribed an antipsychotic medication (operationalised as at least two mentions of the same British National Formulary-class antipsychotic at least one month apart, detected via natural language processing (1)) between 2013-01-01 and 2021-04-30 (2).

We established clozapine use via a gold-standard method, as follows. In the UK, clozapine dispensing occurs in secondary care, and follows registration to a national clozapine registry. We matched CPFTRD records with local records in CPFT that mirror the relevant national clozapine registry for CPFT patients.

The inclusion criteria for the main analysis were (a) for the "ever clozapine" group: being registered with the CPFT clozapine registry during the study period (as above), or (b) for the "ever other antipsychotic" group: use of an antipsychotic within the study period, as detected by natural language processing as above, minus any patients who were in the "ever clozapine" group. Patients were excluded if they had opted out of having their data used, de-identified, for research. In addition, we repeated the analysis restricting the sample to "current" patients: (a) "current clozapine" group: those registered with the clozapine registry on 2021-04-30, and (b) "current other antipsychotic" group: those prescribed an antipsychotic medication between 2019-05-01 and 2021-04-03, minus those in the "current clozapine" group.

ICD-10 psychiatric diagnoses assigned by clinicians were used for a sensitivity analysis of people who had been given a diagnosis of class F20* (schizophrenia).

Linkage to COVID-19 test data was obtained, in de-identified fashion, via the EpiCov project (Cambridge University Hospitals NHS Foundation Trust, CUH) (CPFT project reference M00997). Linkage was achieved as follows: (1) CPFT and CUH agreed a study-specific secret cryptographic hash key, never known to the analysing researchers; (2) for relevant patients in CPFT (selected as above), NHS numbers were hashed to create a study-specific ID (SID) or pseudonym, in automated fashion without humans viewing identifiable information; (3) CPFT passed those SIDs to CUH, without any other data; (4) CUH hashed its known NHS numbers in automated fashion using the same hash key and selected the subset of patients who overlapped by SID; (5) CUH transmitted pseudonymised COVID-19 test data to CPFT for overlapping patients, accompanied by their SID; (6) CPFT linked relevant de-identified patient-level data (antipsychotic use, age, etc.) to COVID-19 data (tests, results) based on the SID, creating a limited and study-specific research database; (7) only then were researchers granted access to that de-identified linked database.

Outcomes were: tested for COVID-19 via polymerase chain reaction (PCR) (binary); tested positive for COVID-19 by PCR (binary). The primary predictor was the use of clozapine (binary). Covariates were: age (continuous, in years); sex (binary); ethnicity (binary: white versus non-white); presence of a coded schizophrenia diagnosis (binary). Associations were tested through logistic regression, and odds ratios (ORs) calculated as the exponentiated coefficients for each predictor.

## Supplementary Table 1: Were clozapine-treated patients more likely to be tested for COVID-19, as compared with other antipsychotic-treated patients?

|  | | | | **OR (95% CI) for having a COVID-19 test** | | |
| --- | --- | --- | --- | --- | --- | --- |
|  | **Total sample, N** | **Clozapine treatment, N (% of total sample)** | **Had COVID-19 test, N (% of total sample)** | **Unadjusted analysis** | **Adjusted for age, sex, and ethnicity** | **Additional adjustment for F20x diagnosis** |
| **Ever on clozapine vs ever on another antipsychotic (Jan 2013–Apr 2021)** | 13,726 | 778 (6%) | 1,831 (13%) | 1.95 (1.67-2.28)  p < 2×10^–16^ | 1.99 (1.70–2.33)  p < 2×10^–16^ | 1.32 (1.10–1.59)  p = 0.002 |
| **Currently on clozapine versus currently on another antipsychotic (May 2019–Apr 2021)** | 13,268 | 544 (4%) | 1,823 (14%) | 1.69 (1.36–2.08)  p = 1.48×10^–6^ | 1.72 (1.38–2.13)  p = 7.46×10^–7^ | 1.05 (0.82–1.33)  p = 0.71 |

## Supplementary Table 2: If tested for COVID-19, were clozapine-treated patients more likely to test positive compared with other antipsychotic-treated patients?

|  | | | | **OR (95% CI) for positive COVID-19 test** | | |
| --- | --- | --- | --- | --- | --- | --- |
|  | **Total sample, N** | **Clozapine treatment, N (% of total sample)** | **Had positive Covid test, N (% of total sample)** | **Unadjusted analysis** | **Adjusted for age, sex, and ethnicity** | **Additional adjustment for F20x diagnosis** |
| **Ever on clozapine vs ever on another antipsychotic (and had a COVID-19 test)** | 1,943 | 233 (12%) | 138 (7%) | 0.96 (0.54–1.60)  p = 0.88 | 1.22 (0.68–2.07)  p = 0.48 | 1.08 (0.57–1.94)  p = 0.81 |
| **Currently on clozapine versus currently on another antipsychotic (and had a COVID-19 test)** | 1,823 | 113 (6%) | 130 (7%) | 0.99 (0.44–1.96)  p = 0.98 | 1.22 (0.53–2.45)  p = 0.61 | 1.06 (0.44–2.28)  p = 0.88 |

## Supplementary Table 3: Were clozapine-treated patients more likely to test positive compared with other antipsychotic-treated patients, irrespective of testing rates?

|  | | | | **OR (95% CI) for positive COVID-19 test** | | |
| --- | --- | --- | --- | --- | --- | --- |
|  | **Total sample, N** | **Clozapine treatment, N (% of total sample)** | **Had positive COVID-19 test, N (% of total sample)** | **Unadjusted analysis** | **Adjusted for age, sex, and ethnicity** | **Additional adjustment for F20x diagnosis** |
| **Ever on clozapine vs ever on another antipsychotic (Jan 2013–Apr 2021)** | 13,726 | 778 (6%) | 138 (1%) | 1.68 (0.95–2.75)  p = 0.05 | 2.09 (1.18–3.48)  p = 0.00713 | 1.26 (0.67–2.26)  p = 0.46 |
| **Currently on clozapine versus currently on another antipsychotic (May 2019–Apr 2021)** | 13,268 | 544 (4%) | 130 (1%) | 1.54 (0.69–2.97)  p = 0.24 | 2.0 (0.88–3.91)  p = 0.06 | 1.10 (0.46–2.33)  p = 0.82 |

## Supplementary References

1. Cardinal RN, Savulich G, Mann LM, Fernández-Egea E. Association between antipsychotic/antidepressant drug treatments and hospital admissions in schizophrenia assessed using a mental health case register. npj Schizophrenia. 2015; 1(1): 1-7.

2. Cardinal RN. Clinical records anonymisation and text extraction (CRATE): an open-source software system. BMC Medical Informatics and Decision Making. 2017; 17(1): 50.
